# Period Poverty and Barriers to Menstrual Health Equity in U.S. Menstruating College Students: A Scoping Review

**DOI:** 10.3390/ijerph22040619

**Published:** 2025-04-16

**Authors:** Lea Sacca, Diana Lobaina, Sara Burgoa, Vama Jhumkhawala, Meera Rao, Goodness Okwaraji, Yasmine Zerrouki, Joshua Sohmer, Michelle Knecht, Maria C. Mejia, Panagiota Kitsantas

**Affiliations:** 1 Department of Population Health and Social Medicine, Charles E. Schmidt College of Medicine, Florida Atlantic University, 777 Glades Road, Boca Raton, FL 33431, USA; dlobaina2021@health.fau.edu (D.L.); sburgoa2022@health.fau.edu (S.B.); vjhumkhawala2022@health.fau.edu (V.J.); mrao2022@health.fau.edu (M.R.); gokwaraji2018@health.fau.edu (G.O.); yzerrouki2016@health.fau.edu (Y.Z.); jsohmer2022@health.fau.edu (J.S.); kebam@health.fau.edu (M.K.); mejiam@health.fau.edu (M.C.M.); pkitsanta@health.fau.edu (P.K.); 2 Department of Health Administration and Policy, George Mason University, 4400 University Drive, Fairfax, VA 22030, USA

**Keywords:** period poverty, menstruating individuals, college students, menstrual equity, United States

## Abstract

Objectives: This scoping review aims to fill research gaps by exploring four guiding research questions. First, we aim to understand the major barriers encountered by U.S. menstruating college students in accessing menstrual health products at their educational institutions, households, and community. Second, we aim to identify social determinants of health (SDoH) that significantly influence and contribute to menstrual health inequities experienced by U.S. college students. Third, we aim to explore the measurement tools that have been used to assess menstrual health inequities experienced by U.S. menstruating college students. Lastly, we aim to determine the lessons learned and recommendations to improve overall menstrual health outcomes in U.S. menstruating college students. Study Design: This scoping review followed the Arksey and O’Malley framework (2005) and incorporated recommendations from the Joanna Briggs Institute (JBI) for the extraction, analysis, and presentation of results. Methods: The four databases searched were PubMed, Embase (Ovid), Web of Science, and Cochrane Library (Medline). Included articles were (1) published between 2000 and 2023, (2) focused on menstruating college students in the United States, (3) addressed menstrual health, menstrual equity, and period poverty in college campuses in the U.S., and/or (4) explored challenges encountered by menstruating college students in accessing menstrual health products. Results: A total of seven studies were retained for assessment. The most frequently cited Healthy People 2030 categories were economic stability (*n* = 5) and social and community context (*n* = 5). The most-cited barriers were sorted into the “Financial Constraints and Accessibility” (*n* = 13) theme category. Conclusions: Our study highlights practical applications and several recommendations for the future design, adoption, implementation, and evaluation of effective evidence-based interventions to address period poverty and subsequent barriers imposed by menstruating college students specific SDoH. More research is needed to further explore the health implications of menstrual health on mental, physical, and socioeconomic outcomes of menstruating individuals, particularly young adults experiencing different challenges as they venture into college and build their careers.

## 1. Background

Every month, around 1.8 billion people menstruate worldwide [1]. Menstruating individuals comprise 26% of the global population and menstruate for 50% of their lifetime [1]. Despite being a natural physiologic phenomenon, many faces significant challenges during menstruation, including period poverty and associated issues of safety and dignity [1]. Menstrual health refers to a state of complete physical, mental, and social well-being that goes beyond the absence of disease or infirmity affecting the menstrual cycle [1,2].

On the other hand, period poverty, defined as menstrual inequity in the form of “insufficient access to menstrual products, education, and sanitation facilities”, remains a human rights issue around the world with several ramifications on attaining optimal menstrual health [2]. Health ramifications for adolescent and adult menstruating individuals include poor hygiene and resulting reproductive tract infections due to limited access to appropriate sanitation facilities [3]. For those who are economically vulnerable, transactional sex is often a means to obtaining basic necessities like sanitary pads, which, in turn, increases these individual’s risk of incurring sexually transmitted infections or experiencing sexual and gender-based violence [4]. Period poverty is not limited in its scope to just appropriate sanitation and waste management [5]. Rather, it extends further to cover social stigmas and taboos that contribute to social ramifications like misinformation and negative attitudes towards menstruation [5].

In the United States (U.S.), about 16.9 million individuals who menstruate live in poverty [6]. Unhoused individuals state that, despite the existence of certain shelters and programs that provide free menstrual products, accessing these products is often difficult, and many times, the amount is not sufficient for even one menstrual cycle [7]. As a result, menstruating individuals often must resort to theft or skipping meals to purchase period products [7]. For those already experiencing period poverty beforehand, the COVID-19 pandemic only exacerbated its effects. A 2021 survey at a free clinic in Pittsburg, PA, found that 40% of female respondents had struggled financially at some point in their life to buy period products—a 5% increase from 2018 [6]. Black or Hispanic menstruating individuals were more likely to experience this struggle, which further illustrates how period poverty in the U.S. disproportionately impacts individuals belonging to racial and ethnic minority communities [6,8]. The implications of period poverty extend to educational disruptions as well, with studies showing that menstruation-related issues can lead to school absenteeism, anxiety, and shame among students across racial groups, age, and socioeconomic status [9,10].

While there is abundant evidence of the impact of period poverty on menstruating individuals in the U.S., a vital population to consider is college students who experience period poverty [11,12]. This population tends to have higher rates of depression, anxiety, food insecurity, and housing instability, all which are associated with poor health outcomes [11,12]. In particular, when paired with the shame and stigma that is often associated with menstruation, period poverty and menstrual equity are significant issues to consider in this population [13,14]. A study found that 14.2% of women attending college experienced period poverty, cited as borrowing products, using other materials in place of menstrual products, or going without any products [15]. Factors that may impact this include lack of access to products, menstrual health and hygiene education, and lack of support for menstrual pain and anxiety [16].

Despite the previous literature, the evidence on menstrual health in this population is limited [9,10,11,12,13,14,15,16]. There is a need to explore and understand how menstrual health and menstrual equity influence the physical, social, and mental well-being of U.S. college students [10,11,12,13,14,15]. This scoping review aims to fill these gaps by exploring four guiding research questions. First, we aim to understand the major barriers encountered by U.S. menstruating college students in accessing menstrual health products at their educational institutions, households, and community. Second, we aim to identify social determinants of health (SDoH) that significantly influence and contribute to menstrual health inequities experienced by U.S. college students. Third, we aim to explore the measurement tools that have been used to assess menstrual health inequities experienced by U.S. menstruating college students. Lastly, we aim to determine the lessons learned and recommendations to improve overall menstrual health outcomes in U.S. menstruating college students. Findings from this study will inform the development of future evidence-based interventions aiming to improve overall menstrual health and decrease menstrual inequities experienced by menstruating U.S. college students.

## 2. Methods

The study sections were organized using the PRISMA-ScR (Preferred Reporting Items for Systematic Reviews and Meta-Analysis extension for Scoping Reviews) as a reference checklist. This review followed the Arksey and O’Malley framework (2005) [17]. and incorporated recommendations from the Joanna Briggs Institute (JBI) [18] for the extraction, analysis, and presentation of results in scoping reviews.

### 2.1. Step 1. Formulating Research Questions on Our Topic of Interest

The four guiding research questions for this scoping review were as follows: (1) What are the major barriers encountered by U.S. menstruating college students in accessing menstrual health products in their educational institution, household, and community?; (2) Which SDoH have been reported to exacerbate menstrual health inequities experienced by U.S. menstruating college students?; (3) Which measurement tools have been used to assess menstrual health inequities experienced by U.S. menstruating college students?; (4) What are the lessons learned and recommendations to improve overall menstrual health outcomes in U.S. menstruating college students?

### 2.2. Step 2. Searching for Studies Relevant to Our Inclusion and Exclusion Criteria

The search strategy was formulated with the help of a senior research librarian (MK) (Supplementary File S1). Search terms included the following: period poverty, menstrual health, menstrual equity, hygiene management, United States, females, women, college, students. The four databases searched were PubMed, Embase (Ovid), Web of Science, and Cochrane Library (Medline). Review of the literature and selection of relevant articles from the databases was completed from October 2023 to January 2024. Screening of the articles based on our inclusion and exclusion criteria was carried out by senior author (LS) and co-authors (DL, SB, VJ, MR, GO, YZ, JS) from January 2024 to April 2024. To limit biases throughout the screening process, initial screening of relevant articles was conducted by six co-authors in two groups of two (DL and SB; VJ and MR) and one group of three (GO, YZ, and JS). A second screening of the articles was carried out by the senior author for a final decision on the total number of articles to include in this scoping review.

#### 2.2.1. Inclusion Criteria

**Figure 1:**
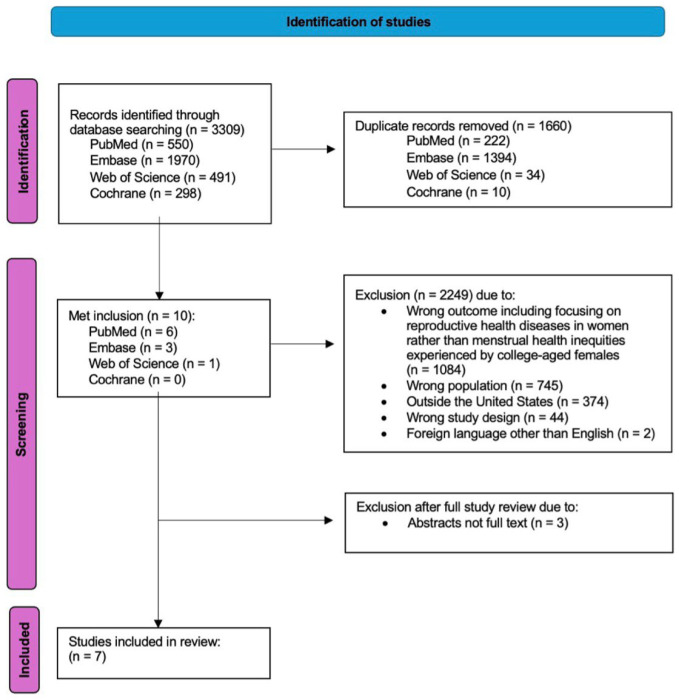
Study selection process based on the PRISMA flowchart.

Included articles were (1) published between 2000 and 2023, (2) focused on menstruating college students in the United States, (3) addressed menstrual health, menstrual equity, and period poverty in college campuses in the U.S., and/or (4) explored challenges encountered by menstruating college students in accessing menstrual health products (Figure 1).

#### 2.2.2. Exclusion Criteria

Excluded articles (1) addressed period poverty or menstrual health in adolescence, (2) were unrelated to menstrual health, menstrual equity, and period poverty in menstruating college students in the U.S., (3) focused on menstrual health and hygiene in general, or (4) generally described menstrual health and menstrual equity rather than focusing on inequities encountered by college students when seeking menstrual health products (Figure 1).

### 2.3. Steps 3, 4, and 5. Selecting Studies Relevant to the Research Questions, and Data Charting and Collation, Summarization, and Reporting of Results

All co-authors (DL, SB, VJ, MR, GO, YZ, JS) extracted and summarized the data from relevant studies. Senior author (LS) reviewed all tabulated data to resolve any discrepancies and to ensure accuracy of data reporting based on findings from included studies. Summary tables included one evidence table (Table 1) describing study characteristics such as primary author/year, study design, sample size, study population, age range, study purpose, type of SDoH, Healthy People (HP) 2030 Category, outcome, type of analysis used in the study, any significant associations, and major findings. Types of SDOH were first listed and then categorized based on Healthy People 2030 into five categories: Economic Stability, Education Access and Quality, Health Care Access and Quality, Neighborhood and Built Environment, and Social and Community Context. The Healthy People 2030 (HP 2030) consists of science-based objectives with targets to monitor progress and motivate and focus action [19]. The HP 2030 first introduced SDOH objectives in 2010, following the World Health Organization’s (WHO) call to address SDOH to maintain health and quality of life, particularly when it comes to the social conditions and environments that are shaped by a wider set of forces and influence behavioral outcomes [19]. Table 2 included a list of barriers that were first classified based on the Socio-Ecological Model (SEM) and then further stratified based on emerging barrier themes that were common across the studies retained for analysis. The SEM framework was selected for barrier stratification as it allows for the exploration of intrapersonal, interpersonal, organizational, environmental, and public policy factors leading to increased rates of period poverty rates in menstruating college students [20].

Table 3 included the type of methodology used to assess the prevalence of period poverty and its impact on physical, mental, and social well-being, whether the tool used to assess impact and/or prevalence of this phenomenon was validated or not, the mode of tool administration, and when relevant, the theoretical framework used and specific constructs measured. Table 4 is a lessons-learned table, carried out through basic qualitative content analysis to identify similar themes to help guide future research directions. The three phases of qualitative content analysis for the results of primary qualitative research described by Elo and Kyngas (2008) [21]. were applied: (i) preparation, (ii) organizing, and (iii) reporting for a structured identification of qualitative themes for synthesis, summary, and emphasis [21].

**Table 1:** Study characteristics.

Primary Author/Year	Study Design	Sample Size	Study Population	Age Range	Study Purpose	Type of SDOH	HP 2030 Category	Outcome	Type of Analysis Used	Major Findings
Cardoso et al., 2021 [15]	Cross-sectional study	*n* = 471	Nationally drawn sample of undergraduate women in the United States	18–24	To examine the frequency of period poverty and its association with poor mental health among university students	Age, Relationship status, Race, Country of Origin, Sexual Orientation, First generation college student status	Education access and quality, Neighborhood and built environment, Economic stability, Social and community context	Mental health (depression)	Multivariable logistic regression	14.2% of the sample experienced period poverty in the past year, 10.0% experienced it every month. Period poverty is significantly associated with depression, on a gradient. Women with monthly period poverty had higher odds of reporting moderate/severe depression.
Dorer, 2021 [22]	Mixed-methods study	*n* = 185	Students from the University of Iowa who have experienced menstruation	18–21	To explore the prevalence and impact of menstrual poverty and stigma among students at the University of Iowa.	Gender identity, Minority status, Family tuition support, FAFSA Qualification	Economic stability and social and community context	Menstrual poverty	Descriptive statistics for survey data; thematic analysis for interview data.	Some students experience menstrual poverty and struggle to afford menstrual products. There is a stigma associated with menstruation, impacting students’ willingness to seek help and discuss their needs.
Foerg, 2021 [23]	Applied research project	Not applicable as it was an advocacy and awareness campaign rather than a traditional study.	Focus on the community of Bowling Green, OH.	N/A	To address period poverty through organizing a menstrual product drive and raising awareness in Bowling Green, OH.	Economic aspects (affordability of menstrual products) and education.	Economic stability and social and community context.	Increased awareness and resource provision for menstrual equity.	N/A	Successful organization of a menstrual product drive and awareness campaign, highlighting the importance of addressing period poverty and menstrual equity.
Gruer et al., 2021 [16]	Qualitative multiple case study	*n* = 20	Students and administrators from four diverse universities in the USA.	N/A	To examine the menstrual equity initiatives across USA universities, identifying obstacles and enabling factors.	Economic access	Economic stability	Implementation and success of menstrual equity initiatives	Thematic analysis	Importance of student and administrative champions in driving initiatives. Need for financial and social support. Challenges in expanding initiatives from pilot to scale.
Mejia, 2022 [24]	Mixed-methods study	*n* = 578	Students at California State University Northridge.	N/A	To analyze the distribution of period products in the San Fernando Valley and its influence on college students.	Age, Neighborhood income, Race, Geographic region	Economic stability, Neighborhood and built environment, Social and community context	Access to menstrual products, period poverty.	Spatial analysis, Descriptive analysis	Inequality in access to period products across different areas, influenced by income and population density.
Rawat et al., 2023 [25]	Qualitative study, focus group discussion	*n* = 32	Students attending Purdue University	18–28	To explore experiences and impacts of a university-wide free menstruation management product policy.	N/A	N/A	Experiences and attitudes regarding free menstrual products and associated cultural impacts.	Thematic analysis	Positive response to free menstrual product access. Suggestions for improving awareness and product quality. Cultural and social implications of menstrual product accessibility in university settings.
Ronning K., 2020 [26]	Mixed-methods study	*n* = 94	Students who menstruate at Arizona State University	18–44	To understand awareness and attitudes toward period poverty among undergraduate menstruators at ASU.	Age, Gender identity, Housing, Race/Ethnicity, Employment status	Economic stability, Neighborhood and built environment, Social and community context	Awareness and attitudes towards period poverty	Quantitative and qualitative analysis	Varied awareness and experiences with period poverty. Importance of accessible menstrual products on campus.
Taylor, 2023 [27]	Mixed-methods study	*n* = 134	Female students living in residence halls at Oregon State University	Predominantly college-aged students.	To understand period poverty and the impact of free menstrual product programs at Oregon State University.	Year in School, Race, Gender Identity	Education access and quality, Social and community context	Perceptions and experiences of period poverty, effects of free menstrual product programs	Descriptive analysis and thematic analysis of focus group discussions	Positive impact of free menstrual product programs but there are suggestions for improvement There are changing attitudes towards menstruation on college campuses

**Table 2:** Identified barriers classified based on the socio-ecological model and theme categories. * Socioecological model level acronyms: Ind (Individual), Inter (Interpersonal), Org (Organizational), Com (Community), and Soc/Pol (Society/Policy).

Article	List of Barriers	Socio-Ecological Model *					Major Barrier Themes						
		Ind	Inter	Org	Com	Soc/Pol	Financial Constraints and Accessibility	Stigma and Cultural Norms	Policy and Institutional Challenges	Education and Awareness	Organizational and Operational Logistics	Product Quality	Environmental Concerns
Cardoso et al., 2021 [15]	Struggle to afford menstrual products	x					x						
	Shame and stigma generally associated with menstruation	x	x		x			x					
	“Tampon tax”					x	x		x				
	Little to no assistance offered to help menstruators afford period products (ex. SNAP or WIC support)				x	x			x				
Dorer, 2021 [22]	Students often face the difficult choice of buying food or menstrual products due to limited financial resources.	x					x						
	There are no guaranteed public services providing free menstrual products outside the public education system.				x								
	Secrecy surrounding the topic of menstruation inhibits open discussion	x	x		x			x					
	Cultural interpretations and myths about menstruation contribute to the stigma		x		x			x					
	Many students have little education about menstruation, leading to misconceptions and inadequate menstrual health management	x		x						x			
	Predominantly male politicians may not prioritize menstrual health needs in policy decisions					x			x				
	Students who cannot afford menstrual products often resort to subpar alternatives	x									x	x	
	Students often depend on friends or family for menstrual products, which can be unreliable and add stress to relationships.		x		x						x		
	Restrictions and embarrassment in accessing menstrual products at school, such as unavailability of products in restrooms or discomfort in asking for them		x	x				x			x		
	Some institutions may provide menstrual products, but they are often of low quality or not well-advertised.			x							x	x	
	Need for more comprehensive sexual education in schools that addresses menstruation openly and honestly				x					x			
	Stress and shame associated with menstrual poverty	x	x		x			x					
	Dispensers for menstrual products that require coins, which can be a barrier for students without spare change.			x			x						
Foerg, 2021 [23]	Continuous lack of access to sanitary products, menstrual hygiene education, and sanitary facilities, especially among low-income individuals			x			x						
	High cost of menstrual products	x			x		x						
	Lack of adequate sex education in schools				x					x			
	Menstruation is often treated as a secret and taboo subject, enforced by education systems and societal norms.	x	x		x			x					
	In schools, boys and girls are often separated during sex education, leading to a lack of understanding about menstruation, particularly for menstruating boys.		x		x					x			
	Insufficient allocation of government resources to address period poverty and a lack of action due to limited awareness and understanding of the issue			x		x			x				
Gruer et al., 2021 [16]	Initiatives often faced resistance from university administration, with concerns about the viability of free product distribution systems, potential for vandalism, misuse, and additional costs.			x					x				
	Difficulties in estimating the real costs of the initiatives. Administrators often overestimated costs, while student budgets typically failed to account for hidden expenses like labor for maintenance and dispenser repairs.			x					x				
	Effective champions were necessary to generate support, navigate bureaucracy, and provide a singular contact point for questions and concerns. The absence of clear leaders or champions often led to fragmented efforts and stagnation.				x	x					x		
	Initiatives faced significant challenges in expanding from small pilots to widespread distribution due to increased funding, labor, and maintenance requirements. Decisions about the source of labor for distributing and maintaining products were crucial.			x							x		
	Initiatives often had to make difficult decisions about product distribution locations due to financial and human resource limitations. This included deciding which bathrooms (female-assigned, male-assigned, gender-neutral) to target for free product distributions.			x							x		
	Ensuring the long-term sustainability of initiatives was a major challenge. It involved securing permanent funding, administrative oversight for stocking and maintenance, and potentially expanding access to more buildings or bathrooms.			x							x		
	Initiatives needed to consider how to incorporate inclusivity, particularly for transgender and non-binary student populations, and avoid gendered language. There was a need to provide menstrual products beyond female-assigned bathrooms		x		x	x							
	Some administration members questioned the need for distributing products in male-assigned locations, impacting the inclusivity and comprehensiveness of the initiatives			x		x			x				
	Initiatives required an understanding of university context and constraints to design effective proposals and gain support from key stakeholders with political, budgetary, and implementation power.			x		x			x				
	Need for initiatives to think beyond immediate implementation and consider longevity and sustainability right from the start			x							x		
Mejia, 2022 [24]	Stores selling period products in the San Fernando Valley (SFV) are not evenly distributed; some areas with smaller populations and higher median incomes have fewer establishments offering these products.				x		x						
	There is a significant price range for period products, with the most expensive options being sustainable products like menstrual cups and menstrual discs. There are also disparities in the availability of these products, with some areas lacking sustainable options entirely.	x		x	x		x						x
	Well-known pharmacies and big-box stores offer the most variety in period products but smaller businesses, particularly those catering to immigrant communities, offer limited options			x	x		x					x	
	Difficulty locating period products when needed	x		x	x		x						
	Inability to afford period products	x					x						
	Students from certain areas felt more comfortable discussing their periods than those from other regions.	x	x		x			x					
Rawat et al., 2023 [25]	Not having a menstrual product when needed is a common experience, often due to the unpredictable nature of periods	x											x
	Financial burden associated with purchasing menstrual products	x											
	Need for higher quality products in dispensers on campus			x								x	
	Concerns about environmental impact of menstrual products	x			x								x
	Many students were unaware of the university’s policy to provide free menstrual products, indicating a need for better communication and promotion of these policies			x						x			
	Discomfort and stigma, particularly in conversations with male community members.	x	x		x			x					
Ronning K., 2020 [26]	Many students were unaware of the on-campus period product initiatives or found them insufficient.			x						x		x	
	The cost of tampons and pads in public restrooms	x					x						
	Those with a strong network of female friends or family members felt more supported, whereas those dependent on males felt less supported		x		x			x					
	Fears that free menstrual products on campus are of lower quality	x		x								x	
	View that managing menstruation as a personal responsibility, indicating a cultural stigma around discussing menstrual health openly	x	x		x			x					
Taylor, 2023 [27]	Economic challenges, with students reusing disposable products or extending the use of products beyond recommended times due to financial constraints.	x					x						
	Lack of confidence and feelings of embarrassment related to menstruation	x	x					x					
	Lack of awareness and promotion about the availability of free period products	x		x			x						
	Discomfort and shame associated with discussing menstruation in their immediate environments.	x			x		x						
	Need for better menstrual education, both formally and informally			x						x			

**Table 3:** Methodology used, mode of administration, and measured constructs.

Primary Author/Year	Type of Methodology Used	Validated	Mode of Administration	Constructs Measured
Cardoso et al., 2021 [15]	Online survey, PHQ-9	Yes (PHQ-9)	Online survey	Period poverty and depression
Dorer, 2021 [22]	Survey and interviews	No	Online survey and in-person interviews	Menstrual poverty, stigma, and related experiences
Foerg, 2021 [23]	Applied research project with advocacy and awareness campaign components	No	Social media campaign, physical collection of menstrual products	Awareness and support for menstrual equity
Gruer et al., 2021 [16]	Qualitative analysis of interviews	No	Interviews	Factors influencing the success and challenges of menstrual equity initiatives
Mejia, 2022 [24]	Spatial analysis, surveys	No	Online survey	Accessibility of menstrual products, period poverty experience
Rawat et al., 2023 [25]	Focus group discussions	No	Virtual	Experiences with and attitudes towards free menstrual products, cultural and social aspects of menstruation
Ronning K., 2020 [26]	Survey and qualitative analysis	No	Online survey	Awareness of period poverty, experiences with menstrual product access
Taylor, 2023 [27]	Survey and focus groups	No	Online survey, in-person focus groups	Experiences with free menstrual products, perceptions of period poverty

**Table 4:** Major themes from lessons learned.

Primary Author/Year	List of Lessons Learned	Major Themes
Cardoso et al., 2021 [15]	High prevalence of period poverty among college-attending women (14.2% in the US in the past year) has significant association with mental health. There is a necessity to improve access to affordable menstrual products and consider period poverty in mental health discussions and policy-making for college students. Future research would be strengthened with the inclusion of appropriate economic measures, incl. food security, for this population There is no standard way to assess period poverty. More research is needed to validate the approach. Future research needed with broader populations of menstruators, including transgender and non-binary populations	**Prevalence and Impact of Period Poverty:** There is a high prevalence of period poverty among college students, which results in significant associations with mental health and attendance issues **Accessibility to Menstrual Products:** There is a dire need to improve access to affordable menstrual products, especially in university settings, and considering period poverty in mental health discussions and policy-making. **Inclusive and Diverse Research:** Future research needs to be more inclusive, considering broader populations including transgender, non-binary, and students of color, as well as the impact of period poverty on university students experiencing homelessness **Stigma and Cultural Attitudes:** There is a need to address menstrual stigma in university settings through education and open discussions. **Policy Development and Advocacy:** Various stakeholders play a critical role in initiating and maintaining menstrual health initiatives and recent policy developments aimed at menstrual equity **Economic and Social Factors:** While economic and social factors play a critical role in period poverty, the issue can affect anyone regardless of their socioeconomic status. **Sustainability and Long-Term Success of Initiatives:** It is critically important to consider sustainability from the onset of any initiative, as well as the challenges in scaling up and ensuring sustainable funding. **Product Availability and Variety:** It is essential to consider the availability and variety of menstrual products in different areas, and the need for sustainable and eco-friendly options. **Healthcare and Medical System Engagement:** Period poverty is also an issue within the medical system, where there is a need for open communication about menstrual pain and symptoms. **Administrative Support and Student Involvement:** Administrative support and student involvement is essential in decision-making regarding menstrual health policies. **Public Awareness and Education:** There is a need for effective promotion strategies to increase awareness and utilization of menstrual health policies, as well as public discussions to reduce stigma. **Impact of Free Menstrual Product Policies:** Providing free menstrual products in educational settings can have positive psychological and cultural effects.
Dorer, 2021 [22]	There is a critical need to address menstrual poverty and stigma in university settings Need for improved access to menstrual products and education about menstruation. While economic and social factors play an important role in menstrual poverty, period poverty can affect anyone	
Foerg, 2021 [23]	Significance of community involvement in addressing menstrual equity. Effectiveness of social media in raising awareness. Importance of practical initiatives like menstrual product drives to directly support those affected by period poverty.	
Gruer et al., 2021 [16]	Critical role of champions in initiating and maintaining initiatives. Importance of social and financial support from key stakeholders with political, budgetary, and implementation power, understanding university context and constraints Importance of use of data and evidence from both student body and other initiatives for a well-conceived and researched proposal. Challenges in scaling up initiatives and ensuring sustainable funding. Immense time commitment required to champion a menstrual health equity initiative as a student could be mitigated by use of student government structures or administrative support Importance of considering how inclusivity could be incorporated into the initiatives, including ensuring access to menstrual products for transgender and non-binary student populations Critical to think about the sustainability of the initiative from the onset to ensure that the initiative is designed in a manner that allows long-term success Additional learning is needed to understand how such initiatives may positively or negatively impact transgender or gender non-binary students, students of color, and those experiencing homelessness while in university	
Mejia, 2022 [24]	Period product availability is higher in densely populated, lower-income areas and commercial zones, with lesser availability in high-income, highly residential areas with less commercial zoning. However, those in higher-income areas generally have better transport options, mitigating the impact of reduced access. The lowest price for tampons and pads was found in dollar stores at $1.25, while the median price in other stores ranged from $4 to $6. Reusable products like menstrual cups and discs, though initially more expensive ($47.00), prove cost-effective over time. Sustainable options are not universally available, with notable absences in areas like Pacoima, Arleta, and Sunland. Well-known pharmacies and big-box stores offered the most variety, while small, locally owned businesses and stores catering to immigrant communities offered less. The greatest product variety was found in neighborhoods like Tujunga, Valley Village, and Van Nuys. A significant portion of menstruating students (37.35%) missed class due to menstrual issues, with financial constraints impacting their ability to purchase period products. There is a noted correlation between access to period products and stigma, with those having less access experiencing more stigma. The study highlighted a need for more inclusive research considering various gender identities and non-traditional menstruators. The majority of respondents were female, indicating a gap in understanding the experiences of other gender identities in relation to menstruation. Recent policy developments aim to address menstrual equity, such as legislation requiring free period products in public restrooms and eliminating the tampon tax. However, more comprehensive measures are needed to ensure access and reduce stigma.	
Rawat et al., 2023 [25]	Students responded favorably to the availability of free menstrual management products at the university. This access not only provided practical benefits but also had broader cultural impacts, highlighting the importance of such policies in educational settings. Nearly half of the study’s participants were unaware of the period policy, partly due to the SARS-CoV-2 pandemic and campus closures, underscoring the need for effective promotion strategies to increase awareness and utilization. Students showed a preference for eco-friendly menstrual products, aligning with broader trends among younger generations favoring environmentally sustainable options. Participants expressed concerns about using product dispensers, citing past experiences with paid, sometimes empty dispensers. Suggestions were made for improved dispenser sanitation and upkeep. Effective advertisement of the period policy and product availability was suggested to be crucial. Methods like social media, magnets, flyers, and strategic placement in high-traffic areas could help increase policy awareness and usage. Openly discussing menstruation management in public spaces can help reduce the stigma around menstruation, improve self-efficacy, and facilitate better access to menstrual products. The study suggests that similar period policies could be effectively implemented in other university settings and community spaces, contributing to increased access and reduced stigma. It’s important for menstruators or those with menstruation experience to be involved in decision-making regarding policies and product selection to ensure the suitability and effectiveness of the provided options. The quality, variety, and brand of menstrual products are crucial for the successful implementation of free menstrual product policies.	
Ronning, 2020 [26]	Participants often experienced period poverty as a personal issue, both at home and on campus. Many were found to use makeshift solutions like toilet paper in emergencies. While some students expressed gratitude for the idea of free period products, others felt guilty about using them, fearing they might deprive someone more in need. This reflects broader societal attitudes towards public goods and social class performance. There is skepticism about the quality of free menstrual products, possibly influenced by long-term branding and marketing strategies by commercial period product companies. The study highlighted the reliance of many menstruators on their mothers or female friends for period products, with those dependent on males feeling less supported. This points to the potential for future research on menstruation stigmatization at a micro, familial level. The movement is key in addressing period poverty, emphasizing the need for accessible, safe, and sustainable period products in higher education and beyond. Providing free period products in public restrooms, similar to toilet paper, is seen as essential for ensuring a dignified and accessible learning experience for all students. The movement should maintain its focus on educated discussions about menstruator choices regarding their bodies and period products, prioritizing health and safety.	
Taylor, 2023 [27]	Although not all students in residence halls experience period poverty, the availability of free period products has been beneficial, reducing stress associated with menstruation and reflecting a body-positive change. Surveys and focus groups indicated a shift in attitudes, with participants feeling more comfortable discussing menstruation, despite some remaining embarrassment. Participants’ experiences highlighted issues in the medical system, such as a lack of open communication about menstrual pain and symptoms, potentially leading to untreated health concerns. The study suggests OSU should extend the availability of period products to all residence hall bathrooms and improve promotion and awareness of these resources. This includes clearer information on where to find these products and their gender-inclusive nature.The University Housing and Dining Services (UHDS) has started ordering period products for residence hall laundry rooms, a move initiated by the Residence Hall Association. The study found a lack of public support from OSU’s administration for the Menstrual Dignity Act. There’s a need for open conversations about menstruation and period poverty to increase awareness and support. Future studies should expand beyond residence halls, encompassing a broader student population. Including administrative perspectives and cost analysis in future research could provide a more comprehensive understanding of the program’s impact and success.	

## 3. Results

The initial study extraction resulted in 3309 articles from PubMed (*n* = 550), EMBASE (*n* = 1970), Web of Science (*n* = 491), and Cochrane (*n* = 298). A total of seven eligible studies based on our inclusion and exclusion criteria were retained for analysis (Figure 1). Included studies were published between 2020 and 2023. Study designs included mixed-methods studies (*n* = 4), qualitative studies (*n* = 2) and cross-sectional studies (*n* = 1). Study populations included menstruating students from various universities in the United States (*n* = 7) (Table 1).

### 3.1. Major SDoH Factors Influencing Menstrual Health Inequities Classified Based on the Healthy People 2030 (HP2030) Categories

The most frequently cited HP2030 categories encompassed economic stability (*n* = 5), social and community context (*n* = 5), neighborhood and build environment (*n* = 3), and education access and quality (*n* = 1) (Table 1). These findings were identified following co-author classification of SDoH factors first based on the five domains of HP2030 categories and then based on relevant barrier theme categories that were apparent across included studies.

### 3.2. Outcomes of Interest and Major Findings

Studies highlighted several outcomes including mental health, menstrual poverty prevalence, implementation and success of menstrual equity initiatives, access to menstrual products, experiences with free products, cultural impacts, period poverty awareness, and personal experiences [15,16,22,23,24,25,26,27]. Particularly, period poverty was significantly associated with depression and other adverse mental health outcomes resulting from the presence of menstrual stigma [15,22]. Such negative perceptions toward menstrual health hindered college students’ willingness to seek help and discuss physical and mental health needs [22]. While the affordability of products remained a key issue for both individuals and organizations, replicating and expanding access to programs offered valuable support, emphasizing the need for continued financial assistance on a large-scale basis [16,23,25,26,27]. Furthermore, the inequalities were recorded in accessibility of menstrual health products across different geographical areas, which emphasizes disparities in affordability and accessibility of essential needs among many menstruating individuals, particularly those residing in underserved areas or coming from low-income backgrounds [24]. Notably, diverse experiences and varied levels of awareness regarding period poverty were identified, with evidence suggestive of changing attitudes towards menstruation on college campuses [25,26,27]. It was seen that college students with higher levels of knowledge on menstruation and overall well-being in general were more likely to report positive attitudes towards this phenomenon compared to those with lower levels of awareness. They also were more likely to associate negative attitudes with inability to afford menstrual products. These findings paint a comprehensive picture of period poverty’s impact, illuminating its multifaceted nature and its connections to mental health, access, experiences, and attitudes [15,16,22,23,24,25,26,27].

### 3.3. Barriers Imposed by Period Poverty on Menstruating College Students

A total of 50 barriers were reported across the seven studies included in this scoping review and all studies reported at least one barrier [15,16,22,23,24,25,26,27]. Barriers were analyzed at the five levels of the SEM, including the Individual level (*n* = 22), Interpersonal level (*n* = 12), Organizational level (*n* = 21), community level (*n* = 20), and society/policy level (*n* = 7). Barriers were also sorted into seven categories based on major recurring themes identified across studies at various levels of the SEM. Among these themes, most barriers (*n* = 13) sorted into the category of “Financial Constraints and Accessibility”. Other barrier categories included “Stigma and Cultural Norms” (*n* = 9), “Policy and Institutional Challenges” (*n* = 7), “Education and Awareness” (*n* = 5), “Organizational and Operational Logistics” (*n* = 9), “Product Quality” (*n* = 6), and “Environmental Concerns” (*n* = 3) (Table 2).

### 3.4. Methodology Used to Assess Period Poverty Prevalence, Awareness, and Impact

Included studies used a variety of methodologies for data collection purposes. Five of the seven studies utilized online surveys as a major methodology [15,22,24,26,27], two used in-person qualitative interviews [16,22], two conducted focus group discussions [25,27], and one study employed a spatial analysis [24]. While eight of the studies used measurement tools that were not validated, one study implemented the PHQ-9 [15], which is a validated measure for depression, as a part of their online survey. These methodologies were used to measure various constructs, including period poverty experiences (*n* = 5) [22,24,25,26,27], perceptions and attitudes toward period poverty (*n* = 2) [23,25,26,27]. accessibility of period products (*n* = 2) [24,26], depression (*n* = 1) [15], stigma (*n* = 1) [22]. and success of menstrual health equity initiatives and their challenges (*n* = 1) [16] (Table 3).

### 3.5. Lessons Learned

The analysis of the lessons learned across various studies revealed several major themes in the context of period poverty and menstrual health in college settings (Table 4). First, the prevalence and impact of period poverty and its significant associations with mental health and attendance issues among college students calls for the critical need for accessible and affordable menstrual product options in college settings [15,22,24,26,27]. Second, there is a need for studies to encompass broader population groups such as transgender, non-binary, students of color, and those experiencing homelessness [15,16,24]. Third, stigma and cultural barriers should be addressed through education and open discussions in colleges [22,24,25,27]. This includes promoting public awareness and education on menstrual health and advocating for the passing of new menstrual health policies that reflect the perspectives of menstruating college students for positive psychological and socio-cultural effects [24,25,27]. Fourth, the role of various stakeholders in policy development and advocacy for menstrual equity should be enhanced to mitigate the impact of socioeconomic challenges on the prevalence of period poverty and increase the widespread sustainability and long-term success of initiatives [16,24,25,26,27]. Fifth, availability of a variety of menstrual health and hygiene management products with a focus on sustainable and eco-friendly options can alleviate the financial burden of period poverty on the healthcare system, which in turn enables a more effective approach to menstrual pain and symptom management [25,27]. These themes collectively underscore the multifaceted nature of period poverty and menstrual health in college environments [15,16,22,23,24,25,26,27]. They highlight the urgency of addressing this issue through a combination of improved access to menstrual products, inclusive research and policy development, engagement of healthcare systems, and the promotion of cultural and educational initiatives. This approach not only aims associated with period poverty but also seeks to foster a more inclusive, informed, and supportive environment for all students [15,16,22,23,24,25,26,27].

## 4. Discussion

This scoping review aimed to highlight the multi-levels barriers experienced by U.S. menstruating college students when accessing menstrual health products in their surrounding environments, while taking into consideration influential SDoH exacerbating such challenges to achieving menstrual equity in this population group. Findings from this study will inform the dissemination and implementation of future evidence-based interventions designed to improve menstrual health and hygiene management disparities and promote menstrual equity in U.S. college students.

Menstruating college students face significant challenges in affording and accessing menstrual health products [28]. Some factors contributing to menstrual health disparities include being a college student, being unemployed and having no financial support, and being unable to cover expenses related to menstrual health as they interfere with the ability to cover basic need expenses such as food, rent, and housing [9,28,29,30,31,32]. A study examining high school students in St. Louis, Missouri, found that 64% faced difficulties affording menstrual health products at least once yearly despite attending a school offering access to these products [9]. Some of these students exclusively relied on products available at school [9,29]. The COVID-19 pandemic potentially intensified these issues, particularly as it related to income loss or unemployment, as revealed by a 2021 Thinx & PERIOD survey, which found that after the pandemic U.S. college students faced the tough decision of choosing between purchasing menstrual products or essentials like food and clothing [29,30,31,32]. These insecurities are widely apparent among racial and ethnic minorities, especially among Hispanic college students coming from low-income families and residing in underserved communities [28,29,30,31,32]. In fact, in-person schooling resumption improved access to menstrual products for students of color [29,30].

In the U.S., it is estimated that the monthly cost of menstrual products is approximately USD 13.25, which adds up to more than USD 6000 before taxes throughout a woman’s reproductive lifespan, which can be a financial and emotional burden for women [33]. While some educational institutions have installed dispensers for free menstrual products in restrooms, there is often a lack of awareness among students regarding these facilities [25]. For instance, Purdue University introduced a policy in 2020 that provided free pads and tampons in all women’s and gender-neutral bathrooms [25]. However, many students were unaware of this policy and referred to these dispensers as “old machines,” indicating a disconnect between the availability of resources and student knowledge of them [25]. Increasing awareness of these policies around college campuses via advertisements, such as signs or flyers throughout campus, social media posts, or emailing to reach students’ attention can help address these discrepancies [25,34]. Students felt mental security and supported by their institution knowing the availability of these products [25,34]. They also allowed for more open and comfortable conversations about menstrual health throughout campus, reducing social stigma [25,34].

Lack of adequate education has contributed to impractical management of the menstrual cycle, misconceptions of menstruation and puberty, and overall feelings of shame, discomfort, and anxiety [25,34,35]. This situation is often worsened when discussions on menstruation are absent in the home environment [35]. Parents are more likely to feel uncomfortable when discussing menstrual health and rely on schools to address such sensitive topics, thus perpetuating an adverse cycle [35]. Furthermore, some schools have restricted teachers and nurses from distributing pain medication, making them feel that they have limited options when helping young female students [35]. As a result, studies have noted that college students in the U.S. who face difficulties affording menstrual products are more likely to experience higher rates of depression later on in life [15,25,34,36]. There is a need to raise awareness about menstrual health in the community to improve the overall well-being of menstruating college students, particularly when it comes to improving college students’ mental health [15].

### 4.1. Implications and Recommendations

Our study highlights practical applications and several recommendations for future design, adoption, implementation, and evaluation of effective evidence-based interventions to address period poverty and subsequent barriers imposed by menstruating college students specific SDoH. For instance, there is an urgent need for college-level initiatives that not only improve knowledge levels on menstrual health, address stigma and negative cultural attitudes towards menstruation, and increase access to affordable menstrual products, but also ensure sustainability of such efforts at the broader community level to enhance product availability and variety in different underserved areas in which college students reside. This requires various stakeholders including public health professionals, physicians, social workers, and policymakers to advocate for new policies allowing for improved accessibility, affordability, and quality of menstrual products at a national level. Long-term interventions can also contribute to better academic performance and less absenteeism in college-aged menstruating individuals. Finally, more research is needed to further explore the health implications of menstrual health on mental, physical, and socioeconomic outcomes of menstruating individuals, particularly young adults experiencing different challenges as they venture into college and build their careers.

### 4.2. Strengths and Limitations

This study has several strengths including having a robust and comprehensive study protocol and methodology which allowed for unbiased and refined coverage of existing literature on period poverty and menstrual health equity among U.S. menstruating students. By identifying the multi-level barriers imposed by SDoH that menstruating college students experience, our findings can inform the development of evidence-based interventions integrating individual and community level strategies to address this public health issue in a holistic manner. This includes providing actionable recommendations for future development and implementation of policies to improve overall menstrual health. Despite the importance of the topic highlighted by this review, several limitations should be taken into consideration. First, a comprehensive search of articles across four databases was carried out after the development and refinement of a detailed search strategy by a senior librarian; however, the review was limited to articles and grey literature (dissertation and thesis projects) in these four databases and did not encompass tracing of reference lists, manual searches of journals, or published scientific reports. Second, the newly emergent terminologies used to refer to menstrual health, menstrual equity, and period poverty created a challenge in the identification of specific terms relevant to our inclusion and exclusion criteria, which in turn might have led to the unintentional omission of search terms. Broader reviews are recommended to account for the limited availability of data and information on period poverty across different sources of the literature. Third, due to the small scope of this review, quality assessment of included studies was not carried out. However, the need to emphasize this important topic to inform the design of future evidence-based interventions is of essence. Future systematic reviews should be carried out to assess the extent of studies exploring the prevalence and impact of period poverty on overall well-being of menstruating individuals and call for action for national efforts to address this public health crisis.

## 5. Conclusions

This scoping review explored barriers encountered by U.S. menstruating college students in accessing menstrual health products, along with the role of SDoH in exacerbating the prevalence of period poverty in this population group. The results may contribute to a broader understanding of factors to consider when designing future menstrual health initiatives in college settings. Lessons learned from previous research studies can guide researchers and community partners to improve the reach and sustainability of menstrual health products and hygiene and sanitation services among U.S. college students.

## Data Availability

Not applicable.

## Supplementary Materials

The following supporting information can be downloaded at: https://www.mdpi.com/article/10.3390/ijerph22040619/s1, Supplementary File S1.
