# Supplementary material for: Period Poverty and Barriers to Menstrual Health Equity in U.S. Menstruating College Students: A Scoping Review
Source: Int J Environ Res Public Health. 2025 Apr 16;22(4):619. doi: 10.3390/ijerph22040619 (PMC12026519; doi:10.3390/ijerph22040619)
Supplement: Supplementary file 1 [file ijerph-22-00619-s001.zip › ijerph-3536507-supplementary.pdf]

## Database: PubMed.gov (Includes MEDLINE)

("menstrual cycle"[Mesh] OR “Menstrual Hygiene Products”[Mesh] OR “Period poverty”[tiab] OR “menstrua*”[tiab] OR “endometrial cycle*”[tiab] OR “Cycle, endometrial”[tiab] OR “Cycles, endometrial”[tiab] OR “Ovarian cycle*”[tiab] OR “Cycle, Ovarian”[tiab] OR “Cycles, Ovarian”[tiab] OR “endometrium cycle”[tiab] OR “female sex cycle”[tiab] OR “ovulatory cycle”[tiab] OR “premenstrual phase”[tiab] OR “catamenia”[tiab] OR “Eumenorrh*”[tiab] OR “Menorrh*”[tiab] OR “Menses”[tiab] OR “Vaginal Tampon*”[tiab] OR “Tampon, Vaginal”[tiab] OR “Tampons, Vaginal”[tiab] OR “Feminine Napkin*”[tiab] OR “Feminine hygiene products”[tiab]) AND (“Universities”[Mesh] OR ((“universit*”[tiab] OR “colleg*”[tiab]) AND (student*[tiab] OR campus*[tiab])))

## Database: Embase.com (Elsevier)

('menstrual cycle'/exp OR 'Feminine Hygiene Product'/exp OR 'Period poverty':ti,ab OR menstrua*:ti,ab OR 'endometrial cycle*':ti,ab OR 'Cycle, endometrial':ti,ab OR 'Cycles, endometrial':ti,ab OR 'Ovarian cycle*':ti,ab OR 'Cycle, Ovarian':ti,ab OR 'Cycles, Ovarian':ti,ab OR 'endometrium cycle':ti,ab OR 'female sex cycle':ti,ab OR 'ovulatory cycle':ti,ab OR 'premenstrual phase':ti,ab OR catamenia:ti,ab OR Eumenorrh*:ti,ab OR Menorrh*:ti,ab OR Menses:ti,ab OR 'Vaginal Tampon*':ti,ab OR 'Tampon, Vaginal':ti,ab OR 'Tampons, Vaginal':ti,ab OR 'Feminine Napkin*':ti,ab OR 'Feminine hygiene products':ti,ab) AND (College/exp OR ‘College Student’/exp OR University/exp OR ‘University Student’/exp OR ((universit*:ti,ab OR colleg*:ti,ab) AND (student*:ti,ab OR campus*:ti,ab)))

## Database: Cochrane Library

('Period poverty':ti,ab OR menstrua*:ti,ab OR 'endometrial cycle*':ti,ab OR 'Cycle, endometrial':ti,ab OR 'Cycles, endometrial':ti,ab OR 'Ovarian cycle*':ti,ab OR 'Cycle, Ovarian':ti,ab OR 'Cycles, Ovarian':ti,ab OR 'endometrium cycle':ti,ab OR 'female sex cycle':ti,ab OR 'ovulatory cycle':ti,ab OR 'premenstrual phase':ti,ab OR catamenia:ti,ab OR Eumenorrh*:ti,ab OR Menorrh*:ti,ab OR Menses:ti,ab OR 'Vaginal Tampon*':ti,ab OR 'Tampon, Vaginal':ti,ab OR 'Tampons, Vaginal':ti,ab OR 'Feminine Napkin*':ti,ab OR 'Feminine hygiene products':ti,ab) AND ((universit*:ti,ab OR colleg*:ti,ab) AND (student*:ti,ab OR campus*:ti,ab))

## Database: Web of Science

((TI="Period poverty" OR AB="Period poverty") OR (TI=menstrua* OR AB=menstrua*) OR (TI="endometrial cycle*" OR AB="endometrial cycle*") OR (TI="Cycle, endometrial" OR AB="Cycle, endometrial") OR (TI="Cycles, endometrial" OR AB="Cycles, endometrial") OR (TI="Ovarian cycle*" OR AB="Ovarian cycle*") OR (TI="Cycle, Ovarian" OR AB="Cycle, Ovarian") OR (TI="Cycles, Ovarian" OR AB="Cycles, Ovarian") OR (TI="endometrium cycle" OR AB="endometrium cycle") OR (TI="female sex cycle" OR AB="female sex cycle") OR (TI="ovulatory cycle" OR AB="ovulatory cycle") OR (TI="premenstrual phase" OR AB="premenstrual phase") OR (TI=catamenia OR AB=catamenia) OR (TI=Eumenorrh* OR AB=Eumenorrh*) OR (TI=Menorrh* OR AB=Menorrh*) OR (TI=Menses OR AB=Menses) OR (TI="Vaginal Tampon*" OR AB="Vaginal Tampon*") OR (TI="Tampon, Vaginal" OR AB="Tampon, Vaginal") OR (TI="Tampons, Vaginal" OR AB="Tampons, Vaginal") OR (TI="Feminine Napkin*" OR AB="Feminine Napkin*") OR (TI="Feminine hygiene products" OR AB="Feminine hygiene products")) AND (((TI=universit* OR AB=universit*) OR (TI=colleg* OR AB=colleg*)) AND ((TI=student* OR AB=student*) OR (TI=campus* OR AB=campus*)))
